# Clinical diagnosis of presumed SOX2 gonadosomatic mosaicism

**DOI:** 10.1080/13816810.2021.1888127

**Published:** 2021-03-15

**Authors:** Malena Daich Varela, Robert B. Hufnagel, Bin Guan, Delphine Blain, Julie C. Sapp, Andrea L. Gropman, Ramakrishna Alur, Jennifer J. Johnston, Leslie G. Biesecker, Brian P. Brooks

**Affiliations:** a Ophthalmic Genetics and Visual Function Branch, National Eye Institute, National Institutes of Health (NIH), Bethesda, Maryland, USA; b Medical Genomics and Metabolic Genetics Branch, National Human Genome Research Institute, NIH, Bethesda, Maryland, USA; c Division of Neurogenetics and Developmental Pediatrics, Children’s National Hospital, Washington, District of Columbia, USA; d Department of Neurology, George Washington University, Washington, District of Columbia, USA

**Keywords:** SOX2, gonadosomatic, mosaicism, microphthalmia, transillumination

## Abstract

**Purpose:**

To describe a family with presumed *SOX2* gonadosomatic mosaicism diagnosed upon ophthalmic examination of the proband’s mother.

**Methods:**

The family underwent comprehensive ophthalmic and physical examination. Variant detection was performed using trio exome analysis on peripheral leukocyte DNA from blood and saliva samples. Variant segregation analysis was performed using a custom panel NGS sequencing. An identified variant in the *SOX2* gene was confirmed in the proband by Sanger sequencing.

**Results:**

We report an individual with bilateral microphthalmia, developmental delay, hearing loss, and dysmorphic features. Her mother was found to have asymptomatic *forme fruste* uveal coloboma affecting her anterior segment. Her father, aunt, and sisters were unaffected. Trio exome sequence analysis showed an apparent *de novo* heterozygous deletion in the proband, NM_003106.3:c.70_89del, NP_003097.1:p. (Asn24Argfs*65), classified as pathogenic. Testing of the other family members’ peripheral blood and saliva was negative for this variant. The iris transillumination abnormalities in the proband’s mother supports a gonadosomatic mosaicism scenario.

**Conclusions:**

The results from this family underscore the importance of performing detailed evaluations of the parents of apparently sporadically affected individuals with heritable ophthalmic disorders. The identification of mildly affected individuals could substantially alter recurrence risks.

## Introduction

Microphthalmia (OMIM #309700) is a developmental malformation in which the eyes are small (axial length <20 mm). It can appear simple (also called pure or primary, where the eye is otherwise structurally normal) or complex (where it is combined with other ocular abnormalities, most frequently microcornea and coloboma) ^(1).^ It has an estimated prevalence of 1 per 7,000 live births (2). Up to 80% of the individuals with microphthalmia have a syndromic form, associated with nonocular malformations (3). Microphthalmia is considered part of a larger phenotypic continuum (i.e., the Microphthalmia-Anophthalmia-Coloboma (MAC) spectrum), where the mildest end is represented by *forme fruste* coloboma. The latter can appear as pigment loss in the inferior quadrant of the iris (sectoral transillumination defects), atypical optic disc cupping, and/or mild, flat choroidal lesions along the six o’clock meridian (4,5). The etiology of most cases of MAC remains unknown, although environmental (infection, nutritional deficiency or drug intake during the first trimester of the pregnancy) and genetic causes have been described (6). Among the last, pathogenic variants in the transcription factor gene *SOX2* have been reported as causative in 10–20% of the individuals with anophthalmia or severe microphthalmia, and in up to 40% of those affected bilaterally (7,8).

## Materials and methods

Participants were recruited and examined under IRB-approved research protocols at the National Institutes of Health (NCT00708955, Evaluation and Treatment Protocol for Potential Research Participants with Ocular Diseases). Complete age-appropriate eye exams were performed.

Leukocyte-derived DNA was isolated from peripheral blood from all six family members; saliva samples from the proband’s mother and aunt were also analyzed (Puregene, Qiagen, Germantown, MD). Trio exome sequencing was performed as described and variants were filtered for apparent *de novo* status (9). Peripheral blood DNA samples from the proband, her two sisters, and her parents and her maternal aunt were also used for a custom panel capture targeting 193 genes followed by Illumina short-read sequencing, as reported (10). A custom NGS analysis pipeline was used for variant calling and filtering as described (11). An expanded panel was used to test the saliva DNA of the mother and the maternal aunt. Briefly, the NEXTFLEX Rapid XP DNA-Seq kit (PerkinElmer) and xGen Lockdown probes (IDTDNA) were used to capture exons and other genomic regions with known or suspected pathogenic variants from a custom 731 genes implicated in eye development or disease. The libraries were then sequenced on an Illumina NextSeq 550 instrument, aligned, variants (including copy number variations) called, annotated, and prioritized through a custom pipeline available on GitHub (https://github.com/Bin-Guan).

The *SOX2* gene region including the variant c.70_89del was then sequenced for the samples from proband, her parents and aunt by Sanger sequencing.

## Clinical report

**Figure 1.**
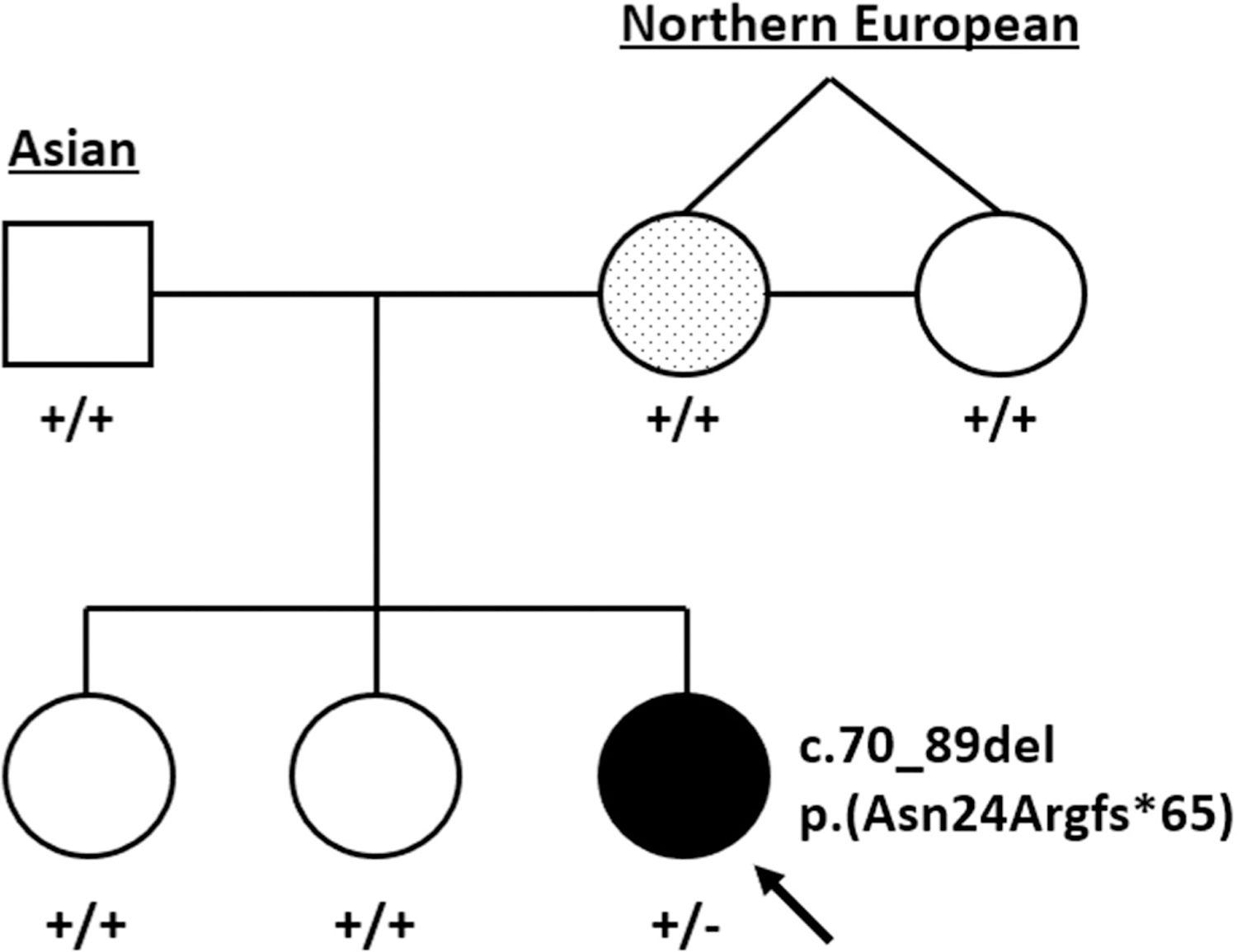
Pedigree representing both parents, maternal aunt (identical twin sister) and children. The proband, indicated by the arrow, has complex microphthalmia and a heterozygous *SOX2* c.70_89del variant. The mother has a presumed *forme fruste* coloboma and a dotted shading to denote suspected mosaicism.

The proband was born at term after an uneventful pregnancy to nonconsanguineous parents of Japanese and northern European descent (Figure 1). Bilateral microphthalmia and coloboma were diagnosed shortly after birth when the parents noted incomplete eye opening. Poor vision in her right eye (OD), mild hyperopia and elevated intraocular pressure in her left eye (OS) were noted by report. She passed the hearing newborn screening.

Developmental delay (speech, fine and gross motor), hypotonia, and crural dystonia were diagnosed around two years of age. Computed tomography of her head demonstrated a small cyst in the corpus callosum and a small right optic nerve when compared to the left. Head circumference, weight and length were within normal limits (10–25th centile) and no tracheoesophageal malformations were found. She had myringotomy tubes placed due to bilateral chronic serous effusions and a left “trigger thumb” surgically released. She had conductive hearing loss in the right ear. At the time of her evaluation at age six years, the child was receiving speech, occupational, and physical therapies. She was taking levodopa/carbidopa for the dystonia and timolol eye drops.

On examination, the proband had minor facial dysmorphic features such as slightly cupped ears and malar flattening, and an irregular capillary vascular malformation at the base of her spine. She had marked weakness in her legs, being able to stand for only a few seconds and take 4–5 steps without assistance. Also, she showed increased tone in her ankles, remarkable dystonic posturing of her feet and 2+ symmetric deep tendon reflexes. She was wearing a prosthetic over the right eye.

**Figure 2.**
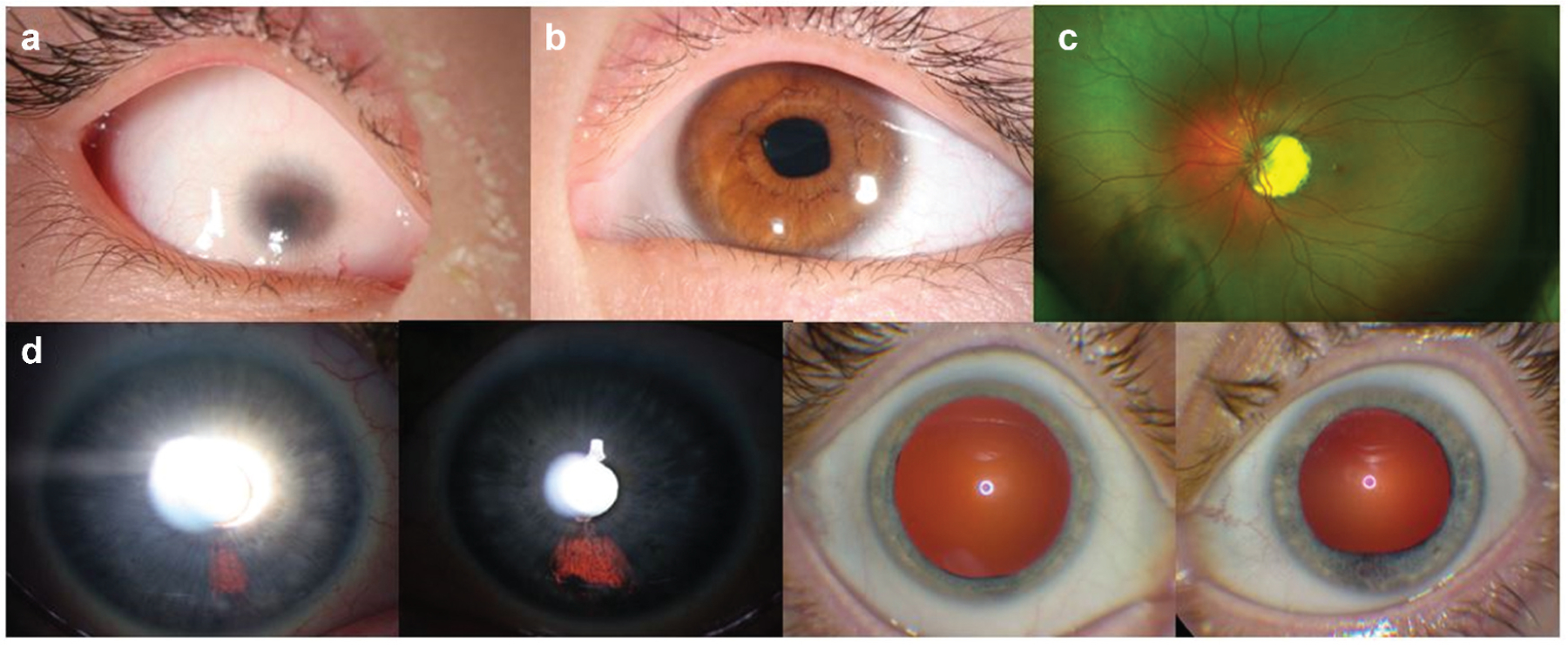
(a) Slit lamp image of the proband’s right eye, showing a vascularized microcornea and a shallow, formed anterior chamber. (b) Proband’s left anterior segment also has microcornea and a mild dyscoria (pupil notch at 3:30 hour). (c) Proband’s posterior segment could only be evaluated OS, showing a uveal coloboma around the optic disc. D) Presumed *forme fruste* coloboma in the proband’s mother, appearing as iris transillumination patches at 5 o’clock OD and 6 o’clock OS, as well as abnormal pupil dilation OS.

Best corrected visual acuity was No Light Perception OD and 20/40 OS, using a mild hyperopic astigmatism correction. Motility was full. External examination demonstrated bilateral microphthalmia, OD>OS. Slit lamp examination showed a right secluded pupil, and a 4 mm vascularized cornea and an irregular left pupil (notch at the 3:30 hour position) and an 8 mm cornea (Figure 2a,b). Her intraocular pressure was normal to palpation OD and 26 mmHg OS. Ocular biometry (IOL Master) demonstrated short axial length (unable to measure OD, 19.93 mm OS) and a steep left cornea (Pentacam: OD, unable; OS, 47.7 D K1, 49.0 D K2) of slightly increased thickness (596 µm). No view of the posterior segment was possible due to the corneal opacity OD. Her left fundus evaluation showed a uveal coloboma surrounding a small optic disc (Figure 2c).

Ophthalmic examinations of the father, two older sisters and aunt (monozygotic twin sister of the proband’s mother) were unremarkable. By contrast, her mother’s evaluation showed bilateral, fairly discrete iris transillumination defects in the inferonasal quadrant, consistent with a *forme fruste* of coloboma (Figure 2d). Visual acuity was 20/20 OD and OS and the remainder of the eye examination was unremarkable.

## Genetic testing results

**Figure 3.**
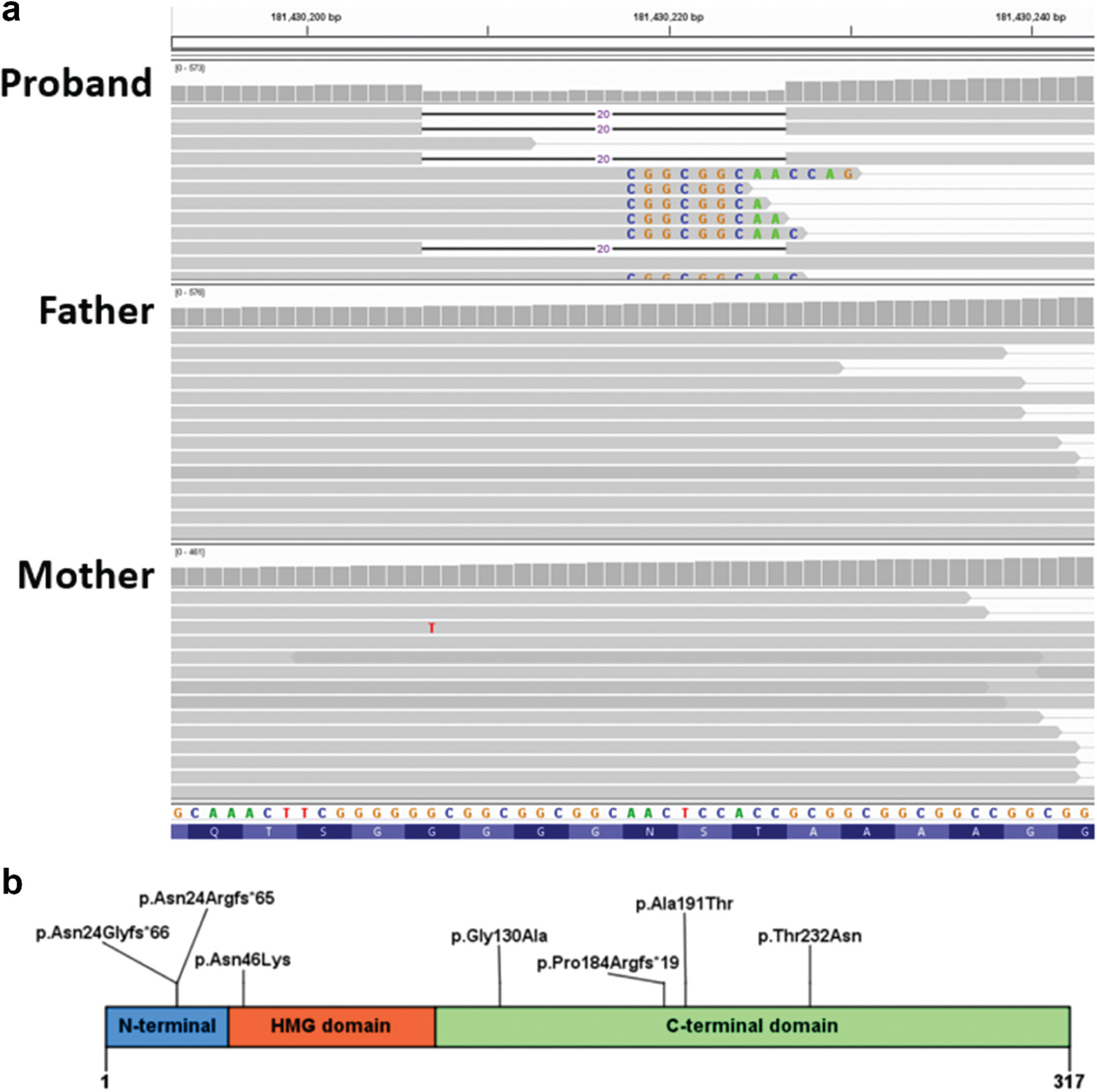
(a) Coverage and alignment tracks in the IGV browser. No read with the 20-bp deletion was found the parents’ data. Sequences of mismatched bases are shown. The reads with mismatched CGGCGGC are expected to contain the 20 nucleotides deletion due to misalignment because of the repeat in the reference sequence. (b) Previously reported predicted SOX2 protein familial variants.

The proband had a normal peripheral blood karyotype (500–650 bands) and a paternally-inherited 2.26 Mb duplication (arr [hg19] 8p23.2(3,674,352–5,938,253)x3 pat; 5 genes) by microarray. This CNV was determined to be non-pathogenic. Trio exome analysis identified an apparently *de novo* heterozygous variant in the *SOX2* gene in the proband (NM 003106:3: c.70_89del, which predicts p.(Asn24Argfs*65)). Panel NGS sequencing of peripheral blood DNA was also performed in the samples from the proband, her two sisters, and her parents (Figure 3a). Biologic parentage was confirmed by segregation of numerous rare variants. The *SOX2* variant was confirmed using Sanger sequencing of the proband’s peripheral blood DNA but was not present in either of her parents’ or the aunt’s peripheral blood DNA samples. This variant was judged to be pathogenic by application of the following criteria: PVS1 (predicted loss of function allele), PS2 (*de novo*, see below), PS4 (previously reported in several individuals with microphthalmia or anophthalmia) and PM2 (not found in gnomAD) (12,13). No other candidate variant was identified in the proband. The mother, aunt and two unaffected siblings, however, carried a variant in the retinoic acid receptor beta (*RARB*) gene (NM_000965.5:c.728 C > G, which predicts p.Thr243Ser) classified as a variant of unknown significance. The absence of any phenotype in the aunt or the two siblings argues against this variant being a confounding cause in the mildly affected mother. Saliva samples from the proband’s mother and aunt also did not show the *SOX2* deletion, with a read depth of 269 in the mother’s sample and 434 on the aunt’s tissue.

Our interpretation of these findings is that the bilateral microphthalmia in the proband was likely caused by her heterozygous *SOX2* variant, which was either (1) *de novo* or (2) inherited from her mother with gonadosomatic mosaicism. The iris transillumination abnormalities in her mother are consistent with somatic mosaicism, despite no *SOX2* variant being observed in her peripheral blood or saliva DNA with 259X NGS read depth at the center of the deletion for the peripheral blood sample (Figure 3a).

## Discussion

*SOX* (sex determining region Y-box) genes represent an important, diverse, and conserved group of developmental transcription factors (14). This family is composed of 20 tissue-specific transcription factors that regulate gene expression during early developmental stages. *SOX2* was described and mapped in 1994, where it was associated with neuronal development (15). Subsequently, a broader function regulating development and cell differentiation in embryonic stem cells was described (16).

In 2003, Fantes et al reported that four individuals within a cohort of 35 with anophthalmia (11%) had *de novo* nonsense (presumed loss-of-function) variants in *SOX2* (17). Several reports followed this one, describing *SOX2* variants in individuals with milder ophthalmic phenotypes such as microphthalmia and uveal coloboma (18). These occurrences were largely due to *de novo* or apparently *de novo* mutation events. In 2006, Faivre et al described a nonconsanguineous family with two of six offspring (one female aborted fetus and one daughter) presenting with clinical anophthalmia (19). The phenotypically normal mother and the daughter had the same heterozygous variant in *SOX2* in blood and buccal specimens. Importantly, the same haplotype present in the mother and the affected child was also present in an unaffected brother who did not have the variant in blood and buccal swabs, consistent with maternal gonadal mosaicism. There were no apparent phenotypic manifestations of *SOX2*-related microphthalmia in the mother.

Mosaicism refers to a phenomenon in which a mutational event occurs post-conceptually, resulting in two or more different cell populations within the same individual (20). Depending on the timing of the post-zygotic mutation, mutant cells can be observed in different patterns. If the mutation occurs before left-right differentiation (in human, exact date unknown, but likely before five weeks of gestation; in mouse, around day 8 post-fertilization) (21), mutant cells populate both sides of the embryo, including the gonads. Mutations that happen after this point are typically unilateral and may show a striking respect for the midline when manifest on the skin. Another embryologic milestone is the differentiation of the primordial germ cells, which occurs by the 24th embryonic day in humans. If a mutation occurs after this point, it usually affects either the somatic or germinal lineage, not both. If the mosaicism hypothesis were correct in the family described here, the maternal variant in *SOX2* must have occurred before the differentiation of the primordial germ cells (gonads and eyes affected), and even before left-right differentiation, (both eyes involved), but after or as a cause of the twinning event (only one twin affected) (22).

The *SOX2* deletion found in the proband is the most commonly reported variant in this gene, accounting for up to 20% of *SOX2*-related occurrences of anophthalmia (23). The deletion from nucleotide 70 to 89 (20 base pairs) is predicted to cause a frame-shift variant upstream of the high-mobility group (HMG) box coding region, producing a premature termination (p. (Asn24Argfs*65)). This predicted truncated protein lacks the HMG and the C-terminal transactivation domains, missing 294 amino acids from the mature protein. As it is a single exon gene, it is not predicted to undergo nonsense-mediated mRNA decay. This alteration is absent in gnomAD (v2.1.1) and classified in ClinVar by three laboratories as pathogenic with no inconsistent assertions (12). The individual reported here not only presented with microphthalmia but also some of the features reported in the *SOX2 *anophthalmia syndrome such as brain malformation, motor abnormalities, and mild facial dysmorphism (6,23,24). The proband’s mother had a milder ocular phenotype and did not have detectable levels of the variant in blood or saliva. Given the read depth of 259 at the center of the deletion in the blood sample, this excludes mosaicism of >1.15% with 95% probability. Digital droplet PCR for rare allele detection was considered but was not technically feasible because sequence repeats in the region of the mutation preclude any PCR amplicons capable of differentiating the reference and the variant sequences, either through a Taqman probe approach or an allele-specific PCR approach. Sequencing of cells of an affected tissue is the gold standard for looking for mosaicism, but this is not possible in our participant (i.e., we cannot biopsy the iris).

We cannot exclude the possibility that these observations represent the coincidence of two, unrelated events. The reported population prevalence of ocular coloboma has a broad range – approximately 0.4 to 4 per 10,000 live births (25,26). The prevalence of iris transillumination defects in the general population has not been studied specifically. However, this has been evaluated in individuals seeking refractive surgery (n = 637 eyes) and in a cohort of patients with glaucoma (n = 24) vs controls (n = 23) (27,28). Either none, diffuse, or radial transillumination patterns were found in these individuals; no focal, *forme fruste* type, transillumination defect were described. Lastly, a review of the electronic medical records from the senior authors’ ophthalmic genetics practice (12/1/2010 to 12/1/2020) – where looking for iris transillumination is a standard part of every exam – there was only one other individual of 1059 unique records with a similar finding. Indeed, this individual was the father of a child with coloboma who also displayed a posterior segment *forme fruste* of coloboma (Brooks, unpublished observation). Although a quantitative prevalence cannot be yet estimated, a circumstantial argument can be made, in which this type of transillumination pattern is not frequent in the general population. Given the rarity of the mother’s phenotypic manifestations, the presence of similar findings in a minority of parents with a child with coloboma, and the lack of other identifiable causative variants, we conclude that one, parsimonious explanation is that she has both somatic and germline mosaicism even in the absence of a confirmatory tissue diagnosis. We cannot exclude the possibility of the mother’s phenotype being caused by other genetic variants currently unknown to cause coloboma and/or environmental factors. In this scenario, we would have to posit the coincidence of two very rare occurrences (*de novo* mutation in *SOX2* and a different cause of sectoral iris transillumination.)

*SOX2*-related eye malformations are reported to be secondary to a *de novo* pathogenic variant in 60% of the cases, whereas approximately 6% are attributed to germline mosaicism, having a presumed “pseudo-autosomal recessive” inheritance pattern (8). Various families are reported in the literature to have transmitted *SOX2* variants to one or more of their children (29,30). These familial occurrences were diagnosed either due to a clear ocular involvement of a parent (irido-chorio-retinal coloboma and anophthalmia) or upon further genetic testing on reportedly unaffected parents. The predicted location of reported family variants within the SOX2 protein is summarized in Figure 3b.

The family reported here represents a scenario in which the presumed transmitting parent had mild ocular features potentially attributable to somatic mosaicism. Because the *forme fruste* of coloboma was asymptomatic and genetic testing was negative in both blood and saliva, some of the reported *SOX2 de novo* and gonadal mosaicism occurrences may indeed have resulted from gonadosomatic mosaicism. Abnormalities such as the one observed in the mother reported here could only be detected with detailed ophthalmic examination and at least some degree of clinical suspicion.

By describing this family we intend to highlight the importance of performing detailed evaluations of the parents of apparently sporadically affected individuals with a MAC spectrum anomaly. The identification of mildly affected parents, coupled with molecular testing for mosaic alterations, could substantially alter recurrence risk counseling, especially pre-conceptually. This may also aid our understanding of *SOX2* inheritance patterns and intrafamilial phenotypic heterogeneity.
